# Exploring the Role of Practice Managers in Dutch Oral Healthcare Practices

**DOI:** 10.1016/j.identj.2024.06.004

**Published:** 2024-07-03

**Authors:** Joost CL den Boer, Wil JM van der Sanden, Katarina Jerković-Ćosić, Josef JM Bruers

**Affiliations:** a Department of Oral Public Health (OPH), Academic Centre for Dentistry Amsterdam (ACTA), University of Amsterdam and Vrije Universiteit Amsterdam, Amsterdam, The Netherlands; b KNMT, Royal Dutch Dental Association, Utrecht, The Netherlands; c Department of Dentistry, Radboud University Medical Centre, Radboud Institute for Health Sciences, Nijmegen, The Netherlands; d HU University of Applied Sciences, Research group Innovation in Preventive Healthcare, Utrecht, The Netherlands

**Keywords:** Practice manager, Corporate dental companies, Oral healthcare practices, The Netherlands, Management tasks, Practice management

## Abstract

**Introduction and aims:**

The practice manager (PM) is a familiar position in Dutch oral healthcare. However, little is known about in which type of practice they work and what their responsibilities are. The aims of this study were to analyse the characteristics of oral healthcare practices that employ a PM and practices that do not, to determine responsibility for tasks described in the PM function description, and to assess to what extent the role of a PM varies between those with an oral healthcare and another background, and across practices with different characteristics.

**Methods:**

At the end of 2022, a questionnaire with questions about the employment of a PM in the practice was presented to 991 randomly selected general dental practitioners. The questions about the tasks of the PM were based on the job description of the PM drawn up by the Royal Dutch Dental Association. Data were analysed using chi-square test, one-way ANOVA, linear regression, and logistic regression analyses.

**Results:**

A PM was employed in the practice of 56% of the general dental practitioners. In many cases, this PM was responsible for a large number of tasks within the sub-areas of care process, human resources, operational policy, and communication. Compared to independent practices, practices affiliated with a corporate dental company often employed a PM and the PMs had a relatively large amount of responsibility.

**Conclusion:**

PMs are now commonly found in Dutch oral healthcare practices, especially in ones that are affiliated with a corporate dental company. The tasks of PMs vary, suggesting an evolving professional profile.

## Introduction

In recent decades, oral healthcare practices (OHPs) in the Netherlands have expanded significantly, marked by the presence of more dental units, a higher number of healthcare providers, and an increased number of patients.^1^ The amount of tasks outside of direct care delivery – for example, organization of care and agenda planning, record keeping, purchasing instruments and materials and human resource management – is higher in large practices, compared to smaller ones.^2^^,^^3^ Traditionally, in Dutch OHPs many of these tasks were conducted by the practice owner, typically a general dental practitioner (GDP).^1^ When GDP-practice owners are occupied with organizational and administrative duties, it comes at the expense of patients’ treatment time. But with the increasing size of dental practices in recent years, practice owners have more and more chosen to hire an employee to perform organizational and administrative tasks. Several factors can be identified for this development, such as the practice owner's desire to focus on patient care, the assessment that the practice's growth is surpassing their capacity to manage additional responsibilities, or the preference to entrust these tasks to someone dedicated to them as their main focus. In the Netherlands, a person engaged in those tasks in OHPs is referred to as a practice manager (PM).

Unlike in the UK, where the role of PM was acknowledged as early as the 1960s, in the Netherlands, this position has only begun to gain significant attention since the beginning of this century, coinciding with a steady increase in the number of PMs over the past decades.^1^^,^^4^^,^^5^ It wasn't until 2009 that the first version of the function description for PM was released. In the 2022 version, by the Royal Dutch Dental Association (KNMT), several tasks regarding human resource management, operational policy, planning, and communication are described.^6^ No specific training is required to perform the position of PM, but there are a number of courses that are tailored to a PM in oral healthcare.^7^ In these courses the focus is on knowledge and skills related to management tasks and not so much on the oral healthcare provided in OHPs.^8^ PMs can have different professional backgrounds. Firstly, they can be managers or office managers who bring to bear their expertise in an OHP. Secondly, they can be existing employees – for example, dental assistants – who specialise in practice management as a career step. The latter seems to apply to a significant part of PMs in Dutch OHPs.^9^ This background can have an impact on the way PMs approach their jobs. After reviewing literature from Scandinavian countries, Kirchhoff and colleagues concluded the way doctors perform management tasks is affected by their medical background.^10^ For nurses, this applied less. German research showed that physicians prefer a PM with a medical background, although this was especially true for physicians who did not work in practice with a PM.^11^

In recent years, the number of OHPs in the Netherlands affiliated with a corporate dental company (CDC) has increased.^1^^,^^12^ Generally, on average these OHPs are larger than their independent counterparts. Consequently, a higher proportion of OHPs affiliated with CDCs can be expected to employ a PM, compared to independent OHPs. The range of tasks of PMs in OHPs affiliated with CDCs is not only determined by the principal GDP but also by policy of the CDC. A study in Australia indicated that CDCs operate according to the four components of ‘McDonaldization’: efficiency, predictability, calculability and control.^13^^,^^14^ In a study of the role of PMs in pharmacies, Scott and colleagues concluded that pharmacist owners have more direct oversight of the processes within the pharmacy than remote owners, which influences the PM's range of duties.^15^ Therefore, it is conceivable that CDCs will have a PM work in their practices more often and will also assign them more tasks.

Two recent studies on collaboration in OHPs in the Netherlands focussed on the role of the practice owner who is also a healthcare provider.^16^^,^^17^ Although PM today is a well-known position in Dutch oral healthcare, little research is available on the range of tasks of PMs and the extent to which their professional background affects this. Even in an international context, studies on PMs in healthcare are scarce, or at least publications in academic journals.^18^ The present study was intended to fill this gap. The main research questions were: What are the characteristics of OHPs that employ a PM, compared to practices that do not? Who is responsible for the execution of the tasks that fit in the function description of PM? And, to what extent does the role of a PM vary between those with a healthcare background and those with a different background, as well as between practices with different characteristics?

## Methods

This study was conducted as a survey among a sample of dentists. The questions for this study were included in a broader questionnaire about collaboration in OHPs in the Netherlands, that was sent to samples of GDPs, dental hygienists, and profylaxis assistants.^17^ This study is based on data obtained only from GDPs.

### Measurement instrument

The research was carried out in the context of Data Stations Project of KNMT, which has been conducting research into various aspects of the professional practice of dentists in the Netherlands since 1995.^1^ The questionnaire developed for this study contained questions about the work situation of the GDP, practice characteristics and the background and activities of the PM. For the purpose of a related study, the questionnaire also contained a number of questions about internal collaboration in the OHP.^17^ The KNMT function description of PM was used as the basis for the questions on the tasks of the PM.^6^ However, the number of tasks in this function description is high and the task descriptions overlap. With the help of an experienced PM, the number of items was reduced to 18, divided over four sub-areas: tasks related to the care process, human resources, operational policy, and communication (for a complete overview, see the questionnaire presented in Supplemental 1). Respondents were asked to indicate per task who is/are responsible for the execution, a GDP, a PM and/or another employee. Regarding the professional background of the PM, respondents were asked whether the PM was trained as and/or had worked as an oral healthcare professional.

### Ethics statement

The research protocol was submitted in advance to the ACTA Institutional Review Board (IRB) under file number 2022-99761. The IRB determined that it complies with the set ethical standards and values and therefore approved the study. All invitations for the survey explicitly stated that participation was voluntary; respondents agreed to participate by completing the questionnaire and sending it by postal mail or digitally.

### Data collection

A random sample of 991 GDPs was drawn from the KNMT dentists’ database. To prevent inviting too many individuals who were no longer working in Dutch oral healthcare, this random sample was drawn from all 9360 GDPs aged 67 years or younger who had a known residential and/or work address in the Netherlands as of January 2022. As usual within the Data Stations Project, the data collection was carried out by an independent agency (KBA Nijmegen). Invitations were sent by postal mail, and questionnaires could be completed on paper or online, using a personal login code that was provided in the invitation letter. In November and December 2022 and January 2023, a total of three reminders were sent to non-respondents. The first reminder was distributed by postal mail and the second and third by e-mail. The invitation and each reminder contained a unique weblink to the survey website. Data collection was terminated by the end of January 2023, after which the independent agency processed the data. The online questionnaires were automatically compiled in a data file, and the answers from the paper questionnaire were manually entered into a comparable file. Both files were then combined into a composite SPSS data file. The data were pseudonymized, ensuring that individual GDPs are not identifiable for the researchers. Some general and professional background characteristics of the participants could also be added to the data, such as gender, year of birth, place and year of graduation, and region of residence. This information was known to KBA Nijmegen.

### Data processing

The questionnaire included three practice characteristics that can be regarded as indicators of the size of the practice: the number of patients who visit the practice at least annually, the number of dental units, and the number of employees. Initially, all three of these characteristics were presented. However, in the multivariate analyses, the high correlation among these three variables – Pearson Correlation Coefficient was 0.747, 0.783, and 0.790, respectively – could potentially cause issues with multicollinearity. Consequently, a decision was made to include only one of these three characteristics in the multivariate analyses, specifically the number of dental units. The number of patients who visit the practice annually was not chosen because a significantly greater number of respondents indicated that they were unable to estimate this compared to the other two characteristics. The number of employees was also not chosen because the PM was considered one of the employees, and the presence of the PM would inherently increase the size of the practice. Moreover, only the number of employees was asked and not the number of hours they worked, which means, for example, two GDPs in one practice can work the same number of hours per week as three GDPs in another. Neither of these drawbacks was associated with the number of dental units. Therefore, this characteristic was chosen as an indicator of the practice size.

In order to compare the tasks performed by PMs, the tasks were aggregated with a weighted approach. This process was carried out both per sub-area and for the overall tasks. The weighting considered whether a PM was solely responsible for a given task or shared that responsibility. Additionally, variations in tasks across different sub-areas and the absence of certain tasks in some practices were taken into account. To address this, a score of 2 was assigned to tasks for which the PM had sole responsibility, a score of 1 for tasks shared with the GDP or another team member, and a score of 0 for tasks the PM was not responsible for. Within each sub-area, scores were summed, and the total was divided by the number of ‘valid’ responses, representing instances where a task was performed within the practice. The same procedure was applied to all involved tasks collectively. This resulted in five variables ranging from 0 (the PM was not responsible for any of the tasks involved) to 2 (the PM was solely responsible for all of the tasks involved).

### Data analysis

The distribution of tasks that could be part of the PM's work activities within OHPs was assessed using descriptive statistics. Additionally, it was investigated whether a correlation exists between the work activities within the PM's domain and certain characteristics of the GDP, PM, and OHP. These analyses were conducted using chi-square test, and one-way ANOVA. Moreover, linear regression analysis and logistic regression analysis were performed to explore multivariate relationships. In all these analyses, all independent variables were included concurrently. The independent variables considered were number of dental units, affiliation of the practice with a CDC, whether the respondent is the practice owner or not, and (if applicable) whether the PM had a background in oral healthcare. In the presentation of the regression analyses, all independent variables are listed, irrespective of their significance in relation to the dependent variable. A significance level of 0.05 was applied to all analyses. The data processing and analysis were carried out using SPSS software, versions 27 and 28.

## Results

### Response

A total of 314 GDPs, representing 31.7% of the 991 invited GDPs and 3.4% of the population of 9360 GDPs, participated in the study by answering the questionnaire completely or largely. Of the participating GDPs, 24 (7.6%) did not work in oral healthcare provision. Additionally, 18 GDPs did not respond to the question regarding the presence of a PM in their practice, leaving a total of 272 GDPs included in the study. Female participants constituted approximately 45.3%, while males made up about 54.7% of the participating GDPs. The average age on January 1, 2023, was 48.0 years. Furthermore, 42.7% of the respondents were located in the west of the Netherlands, 25.5% in the east, 24.3% in the south and 7.5% in the north. It seems that the participants in this study, compared to the population of active GDPs in the Netherlands, were on average somewhat older.^19^ In principle, the questions about the role of the PM were only put to GDPs whose practice had an active PM. However, out of the 153 GDPs to whom this applied, 10 did not respond to the question about the PM's background, and 11 did not answer the questions regarding the PM's tasks.

### PM in practice

A PM was active in the practices of 153 GDPs (56.2%), indicating that this was not the case in the practices of 119 GDPs (43.8%). If a PM was employed, the practice was more often (28.9% vs 3.4%) affiliated with a CDC (Table 1). Moreover, these were relatively larger practices, measured by the number of dental units (average 6.1 vs 3.2). In a logistic regression analysis, which controlled for the other variables, both of these associations remained intact. The results of the logistic regression are included in Supplemental Table S1 in Supplemental File 2.

**Table 1:** OHP characteristics according PM employment status.

	PM employed	No PM employed	Total	
Respondent is practice owner*				
No	56.0%	29.3%	44.4%	
Yes	44.0%	70.7%	55.6%	
*Affiliated with a corporate dental company*†				
No	71.1%	96.6%	82.1%	
Yes	28.9%	3.4%	17.9%	
*Number of dental units*‡				
1-2	9.1%	37.8%	21.7%	
3-5	48.4%	53.8%	50.7%	
6 or more	42.5%	8.4%	27.6%	
*Mean*§	6.1	3.2	4.9	
Standard deviation	3.8	2.0	3.4	
*Total staff size*¶				
5 or less		16.0%	7.0%	
6-10	15.1%	47.0%	29.1%	
11-15	17.8%	20.2%	18.8%	
16-20	19.1%	9.2%	14.8%	
21-25	18.4%	1.7%	11.1%	
26 or more	29.6%	5.9%	18.2%	
*Mean*║	22.9	11.0	17.7	
Standard deviation	14.9	9.5	14.1	
*Number of regular patients*#^,^⁎⁎^,^††				
2500 or less	15.3%	46.2%	30.3%	
2501-5000	37.7%	38.7%	38.2%	
5001-7500	14.1%	11.3%	12.7%	
7501-10,000	18.8%	1.3%	10.3%	
10,001 or more	14.1%	2.5%	8.5%	
*Mean*⁎⁎^,^††	6457.6	3563.0	5054.2	
Standard deviation	4897.5	3127.2	4370.5	
*n*	153	119	272	

OHP, oral healthcare practice; PM, practice manager.

⁎ Chi^2^ (1) = 18.879, *P* < .001.

† Chi^2^ (1) = 29.095, *P* < .001.

‡ Chi^2^ (2) = 53.939, *P* < .001.

§ F (1, 270) = 56.238, *P* < .001.

¶ Chi^2^ (5) = 88.660, *P* < .001.

║ F (1, 269) = 56.778, *P* < .001.

# Chi^2^ (4) = 32.221, *P* < .001.

⁎⁎ Non-response to the question about the number of patients was high, resulting in *n* = 164 (84 PM employed and 80 no PM employed).

†† *F* (1, 163) = 20.194, *P* < .001.

More than half (56.0%) of the GDPs indicated that the PM in their practice had a dental background: 28.0% stated the PM had work experience in oral healthcare, 14.7% cited training in oral healthcare and 13.3% mentioned both. Logistic regression analysis revealed a positive association between a PM's background in oral healthcare and the affiliation of the practice with a CDC (see Supplemental File 2, Table S2)

### Distribution of management tasks

In 16 of the 18 management tasks, more than half of the GDPs indicated that the PM was responsible for their execution, either alone or shared (Table 2). A number of tasks even involved a PM in more than 90% of the cases. Within the sub-area ‘tasks related to the care process’ this concerned ‘supervision compliance with laws and regulations’, within the sub-area ‘human resources’ it concerned ‘coordinating periodic work meetings’, ‘monitoring safety and working conditions in the practice’ and ‘guiding and motivating employees in their daily work’ and within the sub-area ‘operational policy’ to ‘organizing logistical and administrative processes and systems’. The PM was even solely responsible for coordinating periodic work meetings in more than half of the cases.

**Table 2:** Professional(s) responsible for the execution of management tasks in OHPs.

	*Only PM*	*PM and other(s)*	*Only other(s)*
*Tasks related to the care process*			
Ensuring proper handling of complaints	31.4%	55.0%	13.6%
Monitoring efficiency of work processes	31.5%	50.8%	17.7%
Monitoring and controlling the internal quality policy	29.5%	53.5%	17.0%
Supervising compliance with laws and regulations	27.9%	62.5%	9.6%
Patient scheduling	5.1%	37.7%	57.2%
*Human resources*			
Coordinating periodic work meetings	59.1%	32.9%	8.0%
Monitoring safety and working conditions in practice	48.9%	41.3%	9.8%
Coordinating training and education of employees	47.7%	36.4%	15.9%
Execution of performance and annual appraisals	43.2%	44.6%	12.2%
Recruitment and selection	39.7%	45.6%	14.7%
Guiding and motivating employees in their daily work	28.7%	61.8%	9.5%
*Operational policy*			
Preparation of management/financial information for stakeholders	47.6%	27.4%	25.0%
Organizing logistical and administrative processes and systems	46.5%	44.0%	8.5%
Identifying bottlenecks in practice	38.7%	51.2%	10.1%
Managing the purchase of goods and services	14.3%	30.7%	55.0%
*Communication*			
Providing information about practice (e.g. website, social media)	42.7%	31.3%	26.0%
Acting as a point of contact for business relations	37.7%	47.8%	14.5%
Ensuring that educational materials are available to patients	24.0%	38.8%	37.2%

OHP, oral healthcare practices.

*n* = 124-140.

Table 3 provides an overview of the scores on the scales assessing the extent of responsibility of the PM for the execution of management tasks in OHPs, categorized as ‘only PM’, ’PM and other(s)’, and ’only other(s)’. The results of the linear regression to examine the correlation between the scores reflecting the sole or joint responsibility of PMs for the execution of management tasks and the characteristics of GDPs and OHPs are presented in Table 4. This overview shows that PMs had on average more tasks in OHP practices that are affiliated with a CDC than in independent OHPs. All underlying tables, with the results of the linear regression analyses, are included as Supplemental Tables S3 to S7 (Supplemental File 2).

**Table 3:** Responsibility scores for management task execution in OHPs: Assessing the extent of responsibility of the PM on a scale from 0 (others solely responsible) to 2 (PM solely responsible).

	Tasks related to the care process	Human resources	Operational policy	Communication	Total
Mean	1.02	1.33	1.02	1.09	1.15
Median	1.00	1.33	1.00	1.00	1.17
Mode	1.00	1.00	1.00	1.00	1.00
Standard deviation	0.36	0.45	0.50	0.60	0.35

OHP, oral healthcare practices; PM, practice manager.

*n* = 141.

**Table 4:** Associations between scores on scales assessing the extent of responsibility of the PM and GDP and OHP characteristics.

	Tasks related to the care process	Human resources	Operational policy	Communication	Total
PM background in oral healthcare*	0	0	0	0	0
Respondent is practice owner	0	-	0	0	0
Practice affiliated with corporate dental company*	++	++	+	++	++
Number of dental units	0	0	0	0	0

GDP, general dental practitioner; OHP, oral healthcare practices; PM, practice manager*.*

*n* = 137. -: negative association (*P* < .05); 0: no statistically significant association; +: positive association (*P* < .05); ++: positive association (*P* < .01).

⁎ Dichotomised variable (yes vs no).

## Discussion

This study, which focused on the little-researched position of PMs in OHPs, showed that larger practices – with 3 or more dental units – employed one or more PMs in more cases. It was also true that practices that functioned within the larger context of a CDC employed PMs more often than independent practices. This is in line with previous research into corporatization and bureaucratization in healthcare practices.^11^^,^^20^ A possible explanation for this is that in larger organizations there is a greater need for standardization of processes, a need to which PMs can contribute.^16^^,^^21^ Furthermore, we found no relationship between practice size and the number of tasks performed by the PM. This contrasts with Wood's study, which suggested a correlation between practice size and task allocation.^18^

This research demonstrates that PMs generally undertake a diverse range of tasks. Using the KNMT PM job profile, sub-areas have been delineated. However, no pattern emerged between the sub-areas. For example, there was no correlation between the number of communication tasks and the number of operational policy tasks. This might be attributed to the relatively recent establishment of the PM profession in the Netherlands, resulting in its interpretation not yet being clearly defined.

The professional background of PMs varies, a significant proportion have a background in oral healthcare, but a significant proportion do not. This finding substantiates the assertions presented in the professional literature.^9^ The reasons for selecting a PM with expertise in oral healthcare, as opposed to one with a different background, were not addressed in this study. German research indicates that physicians may prefer project managers with a medical background.^11^ This prompts the question of whether the decision to opt for a project manager without training or experience in oral healthcare reflects a substantive choice or is primarily influenced by availability. Further investigation is warranted.

With regarding to the tasks of PMs, 18 management tasks have been identified based on the KNMT PM job profile, categorized into 4 focus areas. Across all focus areas, PMs predominantly carried out the associated tasks, either independently or in collaboration. In particular, a vast majority of PMs were entrusted with sole or shared responsibility for overseeing compliance with laws and regulations, coordinating periodic work meetings, monitoring safety and working conditions in the practice, guiding and motivating employees in their daily tasks, and organizing logistical and administrative processes and systems. While we previously observed that CDC-affiliated practices tend to employ PMs more frequently, it is also evident that within these practices, PMs undertook a wider array of tasks compared to independent practices. This aligns with our expectation, drawn from prior studies indicating that corporate practices prioritize efficiency, predictability, calculability, and control.^13^^,^^14^ Moreover, by reducing the time spent on administrative tasks, practice owners can devote more attention to the efficient delivery of the practice's primary activity, providing oral healthcare. Furthermore, PMs can also play a role in standardizing processes, which is particularly crucial within CDCs.^16^^,^^21^

### Limitations and strengths

The questions for this study were part of a larger questionnaire that also assessed aspects of collaboration between GDPs, dental hygienists, and prophylaxis assistants. However, because the items regarding the role of the PM were placed at the end of the questionnaire, the study experienced attrition and potential attrition bias. Item non-response is a recognized issue in surveys; dropout rates vary depending on the topic, with many respondents ending the questionnaire at a point where the topic changes.^22^, ^23^, ^24^ In this case, the risk of attrition bias should be considered, as practice owners are likely to be more familiar with and interested in the activities of PMs. Nevertheless, this did not appear to have a significant impact, as only two respondents dropped out during the PM questions.

Due to limited space in the questionnaire, we had to make certain decisions regarding the scope of our study. As a result, the study primarily focuses on the dental training and experience of PMs, without delving into any additional courses they may have taken in management tasks. Furthermore, we directed questions about responsibilities within the PM's domain solely to those who currently employ a PM. For OHPs without a PM, there was no information regarding who undertakes these tasks. However, this aspect is noteworthy, as the responsibility may not always fall on the practice owner; it could also be another GDP or employee.

This study has several strengths, including a well-structured questionnaire and a significant number of participants. To the best of our knowledge, this is the first study of its scale to investigate the responsibilities carried out by PMs in OHPs, particularly within the context of the Netherlands. By aligning the tasks with the established job profile of a PM, we systematically mapped the distribution of these responsibilities in a manner that closely mirrors real-world practice.

### Implications for practice

In the Netherlands, the PM profession is relatively new and still evolving.^1^ Despite the existence of a job profile, there appears to be considerable variation in the interpretation of the profession, similar to the situation observed in Germany with PMs in medical practices.^11^ This study reveals that PMs in Dutch OHPs come from diverse backgrounds. While a significant proportion have acquired knowledge of OHP through training or previous work, a notable portion had no prior experience in oral healthcare before taking on the role of PM. Training programs and further education initiatives for PMs primarily focus on acquiring management knowledge and skills, a choice that is justifiable given the breadth of responsibilities entrusted to PMs.

Interestingly, the presence or absence of training and/or work experience in oral healthcare did not exhibit a statistically significant relationship with the number of tasks performed by PMs. Those without a background in oral healthcare were also observed to undertake tasks related to the care process. Moreover, research conducted in the United Kingdom has demonstrated a positive correlation between the presence of a PM in practice and adherence to certain guidelines, thereby influencing the provision of care.^25^ Consequently, gaining deeper insights into the content and processes surrounding the delivery of oral healthcare could prove beneficial for PMs. Therefore, integrating such knowledge more into existing training programs for PMs would be a valuable enhancement.

CDCs have experienced significant growth in recent years.^1^^,^^26^ This study revealed that OHPs affiliated with a CDC differed from independent OHPs in terms of their organizational structure. Not only did CDC-affiliated practices employ PMs more frequently, but they also assigned them more tasks. As CDCs continue to expand, the demand for PMs is likely to increase accordingly. With an adequate supply of PMs, they could potentially assist practicing GDPs and DHs in allocating more time to patient care tasks. This is particularly desirable given the reported shortages of GDPs and dental hygienists.^27^

## Conclusion

The rather new profession of PMs is already frequently employed in Dutch OHPs, particularly in practices affiliated with CDCs. Their range of tasks is diverse, indicating that the professions profile does not seem to have been fully developed yet.

## Conflict of interest

The authors have no conflicting interests to report.

## Appendix Supplementary materials

Image, application 1

Image, application 2

## Footnotes

Supplementary material associated with this article can be found in the online version at doi:10.1016/j.identj.2024.06.004.
