# Supplementary material for: Exploring the Role of Practice Managers in Dutch Oral Healthcare Practices
Source: Int Dent J. 2024 Jul 3;75(1):248–55. doi: 10.1016/j.identj.2024.06.004 (PMC11806326; doi:10.1016/j.identj.2024.06.004)
Supplement: Supplementary file 2 [file mmc2.docx]

**Supplement 2 – Supplementary tables**

| **Table S1 Logistic regression: PM employment status and OHP characteristics** | | | | | |
| --- | --- | --- | --- | --- | --- |
|  | *B* | *SE* | *Beta* | *95%-CI* | *p* |
| intercept | -1.558 | 0.418 | 0.211 |  | 0,000 |
| respondent is practice owner | -0.554 | 0.307 | 0.574 | 0.315 – 1.049 | 0,071 |
| practice affiliated with corporate dental company ^#1^ | 1.862 | 0.578 | 6.438 | 2.075 – 19.980 | 0,001 |
| number of dental units | 0.445 | 0.082 | 1.561 | 1.330 – 1.832 | 0,000 |
| n = 272 | | | | | |
| Nagelkerke R^2^ = 0.396 | | | | | |
| #1 dichotomised variable (yes versus no) | | | | | |

| **Table S2 Logistic regression: training or work experience of PM in oral healthcare and OHP characteristics** | | | | | |
| --- | --- | --- | --- | --- | --- |
|  | *B* | *SE* | *Beta* | *95%-CI* | *p* |
| intercept | 0.261 | 0.415 | 1.299 |  | 0.529 |
| respondent is practice owner | 0.583 | 0.369 | 1.792 | 0.870 – 3.693 | 0.114 |
| practice affiliated with corporate dental company ^#1^ | 0.956 | 0.417 | 2.602 | 1.148 – 5.894 | 0.022 |
| number of dental units | -0.089 | 0.049 | 0.914 | 0.830 – 1.007 | 0.070 |
| n = 140 | | | | | |
| Nagelkerke R^2^ = 0.095 | | | | | |
| #1 dichotomised variable (yes versus no) | | | | | |

| **Table S3 Linear regression: scores on scales assessing the extent of responsibility for tasks related to the care process of the PM and PM and OHP Characteristics** | | | | | |
| --- | --- | --- | --- | --- | --- |
|  | *B* | *SE* | *Beta* | *95%-CI* | *p* |
| intercept | 1.011 | 0.081 |  | 0.851 – 1.171 | < 0.001 |
| PM has background in oral healthcare | 0.096 | 0.064 | 0.132 | -0.030 – 0.222 | 0.134 |
| respondent is practice owner | -0.058 | 0.064 | -0.080 | -0.184 – 0.068 | 0.365 |
| practice affiliated with corporate dental company ^#1^ | 0.115 | 0.070 | 0.145 | -0.024 – 0.254 | 0.104 |
| number of dental units | -0.010 | 0.008 | -0.102 | -0.026 – 0.005 | 0.235 |
| n = 137 | | | | | |
| R^2^ = 0.066 | | | | | |
| #1 dichotomised variable (yes versus no) | | | | | |
| positive association (p < 0.05) | | | | | |

| **Table S4 Linear regression: scores on scales assessing the extent of responsibility for human resources tasks of the PM and PM and OHP Characteristics** | | | | | |
| --- | --- | --- | --- | --- | --- |
|  | *B* | *SE* | *Beta* | *95%-CI* | *p* |
| intercept | 1.347 | 0.095 |  | 1.159 – 1.535 | < 0.001 |
| PM has background in oral healthcare | 0.076 | 0.075 | 0.085 | -0.072 – 0.223 | 0.313 |
| respondent is practice owner | -0.150 | 0.075 | -0.170 | -0.298 – -0.003 | 0.046 |
| practice affiliated with corporate dental company ^#1^ | 0.249 | 0.083 | 0.256 | 0.085 – 0.412 | 0.003 |
| number of dental units | -0.009 | 0.010 | -0.082 | -0.028 – 0.009 | 0.325 |
| n = 137 | | | | | |
| R^2^ = 0.131 | | | | | |
| #1 dichotomised variable (yes versus no) | | | | | |
| negative association (p < 0.05) | | | | | |
| positive association (p < 0.05) | | | | | |

| **Table S5 Linear regression: scores on scales assessing the extent of responsibility for operational policy tasks of the PM and PM and OHP Characteristics** | | | | | |
| --- | --- | --- | --- | --- | --- |
|  | *B* | *SE* | *Beta* | *95%-CI* | *p* |
| intercept | 0.886 | 0.112 |  | 0.665 – 1.107 | < 0.001 |
| PM has background in oral healthcare | 0.013 | 0.088 | 0.014 | -0.160 – 0.187 | 0.878 |
| respondent is practice owner | 0.066 | 0.088 | 0.066 | -0.108 – 0.239 | 0.456 |
| practice affiliated with corporate dental company ^#1^ | 0.242 | 0.097 | 0.222 | 0.050 – 0.434 | 0.014 |
| number of dental units | 0.005 | 0.011 | 0.014 | -0.017 – 0.027 | 0.640 |
| n = 137 | | | | | |
| R^2^ = 0.050 | | | | | |
| #1 dichotomised variable (yes versus no) | | | | | |
| positive association (p < 0.05) | | | | | |

| **Table S6 Linear regression: scores on scales assessing the extent of responsibility for communication tasks of the PM and PM and OHP Characteristics** | | | | | |
| --- | --- | --- | --- | --- | --- |
|  | *B* | *SE* | *Beta* | *95%-CI* | *p* |
| intercept | 1.079 | 0.132 |  | 0.817 – 1.341 | < 0.001 |
| PM has background in oral healthcare | 0.021 | 0.104 | 0.018 | -0.185 – 0.227 | 0.839 |
| respondent is practice owner | 0.088 | 0.104 | 0.073 | -0.118 – 0.294 | 0.401 |
| practice affiliated with corporate dental company ^#1^ | 0.311 | 0.115 | 0.237 | 0.083 – 0.539 | 0.008 |
| number of dental units | -0.021 | 0.013 | --0.133 | -0.047 – 0.006 | 0.122 |
| n = 137 | | | | | |
| R^2^ = 0.073 | | | | | |
| #1 dichotomised variable (yes versus no) | | | | | |
| positive association (p < 0.05) | | | | | |

| **Table S7 Linear regression: scores on scales assessing the extent of responsibility of the PM and PM and OHP Characteristics** | | | | | |
| --- | --- | --- | --- | --- | --- |
|  | *B* | *SE* | *Beta* | *95%-CI* | *p* |
| intercept | 1.141 | 0.074 |  | 0.995 – 1.288 | < 0.001 |
| PM has background in oral healthcare | 0.062 | 0.058 | 0.090 | -0.053 – 0.178 | 0.289 |
| respondent is practice owner | -0.053 | 0.058 | -0.077 | -0.169 – 0.062 | 0.365 |
| practice affiliated with corporate dental company ^#1^ | 0.224 | 0.065 | 0.296 | 0.097 – 0.352 | <0.001 |
| number of dental units | -0.010 | 0.007 | -0.108 | -0.024 – 0.005 | 0.193 |
| n = 137 | | | | | |
| R^2^ = 0.130 | | | | | |
| #1 dichotomised variable (yes versus no) | | | | | |
